# Natural Progression of Left Ventricular Function following Anthracyclines without Cardioprotective Therapy: A Systematic Review and Meta-Analysis

**DOI:** 10.3390/cancers15020512

**Published:** 2023-01-13

**Authors:** Ainsley Ryan Yan Bin Lee, Chun En Yau, Chen Ee Low, Jiaqi Li, Sara Moiz Tyebally, Weiqin Lin, Li-Ling Tan, Chia-Te Liao, Wei-Ting Chang, Matilda Xinwei Lee, Chieh-Yang Koo, Ching-Hui Sia

**Affiliations:** 1 Yong Loo Lin School of Medicine, National University of Singapore, Singapore 117597, Singapore; 2 School of Clinical Medicine, University of Cambridge, Cambridge CB2 0SP, UK; 3 Division of Cardiology, Ng Teng Fong General Hospital, Singapore 609606, Singapore; 4 Department of Cardiology, National University Heart Centre Singapore, Singapore 119074, Singapore; 5 Division of Cardiology, Department of Internal Medicine, Chi Mei Medical Center, Tainan 71004, Taiwan; 6 Department of Public Health, College of Medicine, National Cheng Kung University, Tainan 70101, Taiwan; 7 Studies Coordinating Centre, Research Unit Hypertension and Cardiovascular Epidemiology, KU Leuven Department of Cardiovascular Sciences, University of Leuven, 3000 Leuven, Belgium; 8 Department of Biotechnology, Southern Taiwan University of Science and Technology, Tainan 71005, Taiwan; 9 Department of Haematology-Oncology, National University Cancer Institute, Singapore 117597, Singapore

**Keywords:** anthracyclines, cardiotoxicity, heart failure, chemotherapy toxicity, cardio-oncology, breast cancer, haematological cancer

## Abstract

**Simple Summary:**

Anthracyclines form the backbone of many systemic chemotherapy regimens with great response rates for cancers. Yet, their established dose-limiting cardiotoxic effects can also lead to a reduction in cardiac function and an increased risk of heart failure. This PRISMA-adherent systematic review and meta-analysis of randomised-controlled trials aims to evaluate the progression of cardiac dysfunction and levels of natriuretic peptides, and risk of heart failure in cancer patients receiving anthracyclines. Our review included cohorts which followed patients over two years from the administration of anthracyclines and demonstrated that there were no significant declines compared to after six months. This period would be the most crucial for concurrent cardioprotection to prevent adverse remodelling. We also found the risk of developing significant declines in LVEF occurred in approximately one in six. The confounding effect of receiving concomitant trastuzumab and baseline LVEF was also negligible.

**Abstract:**

Background: Anthracyclines form the backbone of many systemic chemotherapy regimens but are accompanied by dose-limiting cardiotoxicity. We elucidate the progression and severity of cardiac function over time, in the absence of cardioprotection, which less is known about. Methods: This PRISMA-guideline-adherent review was registered on PROSPERO (CRD42022373496). Results: 26 studies met the eligibility criteria including a total of 910 patients. The overall reduction in post-anthracycline pooled mean left ventricular ejection fraction (LVEF) in placebo arms of the included randomised-controlled trials was 4.5% (95% CI, 2.6 to 6.4). The trend in LVEF showed a progressive decline until approximately 180 days, after which there was no significant change. Those receiving a cumulative anthracycline dose of 300 mg/m2 experienced a more profound reduction. The overall pooled risk of a 10% absolute decline in LVEF from baseline, or a decline to an LVEF below 50%, was 17% (95% CI: 11 to 24; I2 = 71%). Sensitivity analyses of baseline LVEF and trastuzumab treatment status did not yield significant differences. Conclusion: While the mean LVEF decline in patients without cardioprotective therapy was clinically small, a vulnerable subset experienced significant impairment. Further research to best identify those who benefit most from cardioprotective therapies when receiving anthracyclines is required.

## 1. Introduction

In the setting of advanced or disseminated cancers including breast and haematological cancers, systemic chemotherapy involving anthracyclines has been at the forefront of management for years [1,2,3]. However, the administration of anthracyclines is often limited by the dose-dependent risk of cardiotoxicity [4]. Anthracyclines have been associated with cardiac dysfunction or heart failure in up to 20% of all patients [5].

Cardiac dysfunction and heart failure is a critical adverse event following anthracycline therapy, which can result in the interruption of cancer therapy, potential severe adverse cardiac events and even death [6,7]. Thus, this has necessitated the identification and administration of interventions for prophylaxis or the early prevention of anthracycline-induced cardiac dysfunction [8]. However, results from recent trials remain largely equivocal. Moreover, most studies reporting the incidence and risk of cardiac dysfunction in patients receiving anthracycline therapies were older studies and did not use current clinical practices [9,10]. As such, it remains unclear what effects anthracyclines have on the natural progression in cardiac function in current clinical practice.

To further understand and describe the temporal effect of anthracyclines on cardiac function, we performed a systemic review with network meta-analysis including on adult cancer patients receiving anthracyclines without cardioprotective therapy. We hypothesized that the effect of anthracyclines on cardiac dysfunction in current clinical practice was lower than previously reported in older studies. Our aim was to describe the change in cardiac function through left ventricular ejection fraction and natriuretic peptides over time. Our other aims were to identify risk factors predictive of significant cardiac dysfunction.

## 2. Methodology

This systematic review with network meta-analysis was reported in accordance with the PRISMA statement for systematic reviews [11]. The protocol was registered on PROSPERO (registration: CRD42022373496).

### 2.1. Selection of Studies

We searched Medline, Embase, and Cochrane Central Register of Controlled Trials from database inception until 15 September 2022. Our search combined an exhaustive list of concepts, language, and keywords for randomised controlled trials, cardiotoxicity, and anthracyclines (Table S1). We also searched reference lists of relevant systematic reviews and clinical guidelines.

Two authors (ARYBL and JL) independently selected eligible studies based first on the titles and abstracts, followed by full text articles, with conflicts resolved by a third author (CHS). We included randomised-controlled trials with adult participants, defined as participants of at least 18 years of age, with a diagnosis of any solid or haematological cancer for which they were receiving anthracyclines, and involving at least one arm of the study administering pharmacotherapy for the prevention of long-term cardiac dysfunction compared to a cohort receiving standard care or a placebo with no cardioprotective therapy.

### 2.2. Data Extraction

Data of each included study was extracted by at least two authors independently (ARYBL and JL) and checked for quality at the end of the extraction phase. Outcomes of interest related to measures of systolic dysfunction, including left ventricular ejection fraction and pro-brain natriuretic peptide or brain natriuretic peptide (natriuretic peptides). We defined a clinically relevant decrease in LVEF as a 10% decline in LVEF from baseline to a value below 50% where possible [12]. Where studies defined a different definition of significant LVEF decline, this definition was extracted and reported.

### 2.3. Quality Assessment and Certainty of Evidence

The Cochrane Risk of Bias 2.0 tool was used to assess the quality of included studies. Each study was assessed by two authors independently with discrepancies resolved by consensus, with results reported in Figure S1 [13].

### 2.4. Data Analysis

The extracted data were quantitatively pooled and analysed in R Version 4.2.1 using the meta and metafor packages. In studies without standard deviations (SDs), confidence intervals (CIs) were converted to SDs. In studies without relevant baseline data, the simple analysis of the final values method was used. Studies were pooled for meta-analysis using standardised mean differences (SMD) and the random-effects model. Between-study heterogeneity was represented by I2 and τ2 statistics. I2 of <30% indicated low heterogeneity between studies, 30% to 60% showed moderate heterogeneity, and >60% indicated substantial heterogeneity. Unless specified otherwise, we considered a two-sided *p* value of < 0.05 to be statistically significant.

We performed pre-planned subgroup analysis according to key study characteristics. So that the effect of anthracyclines could be isolated, we only included studies in which all patients received anthracyclines. To account for different anthracycline toxicities, conversion to doxorubicin equivalent doses was performed [14]. We also identified studies in which more than 10% of patients received trastuzumab during the period of observation and repeated analysis excluding these studies. Additionally, leave-one-out analysis, outlier analysis and repetition of the primary analysis with the common-effects rather than random-effects model was performed and presented.

## 3. Results

### 3.1. Results of the Literature Search

**Figure 1:**
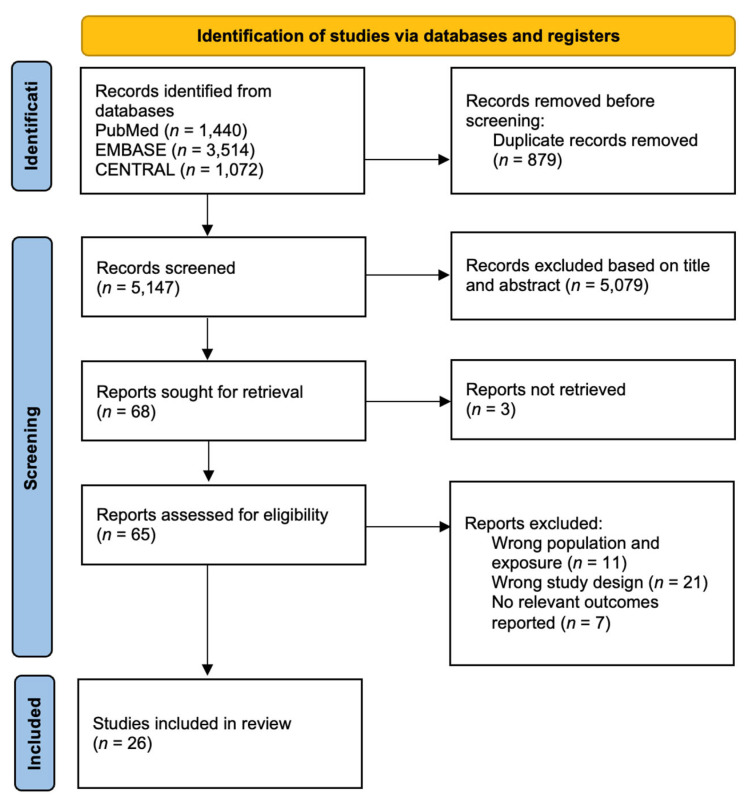
PRISMA flowchart.

From 5147 unique records identified from our literature search of PubMed, EMBASE, CENTRAL and Scopus, a total of 26 [15,16,17,18,19,20,21,22,23,24,25,26,27,28,29,30,31,32,33,34,35,36,37,38,39,40] studies met our inclusion criteria. The results of our search are presented in Figure 1. The key participant, trial and treatment characteristics of each study are detailed in Table S2 with details of the outcome assessment in Table S3.

All studies included participants with a baseline mean LVEF above 50%. The highest mean baseline LVEF was 69.7% [31] and the lowest was 54.9% [21,37]. A total of 9 out of 26 studies had a baseline mean LVEF above 65.0% [18,19,22,27,30,31,33,38,40], 13 out of 26 studies were between 60.0% to 65.0% [15,16,17,20,23,24,25,26,28,32,34,36,39], 2 out of 26 studies between 55.0% to 60.0% [29,35] and 2 out of 26 studies between 50.0% and 55.0% [21,37]. Anthracyclines administered to cancer patients included doxorubicin, epirubicin, idarubicin and daunorubicin.

### 3.2. Left Ventricular Ejection Fraction

**Figure 2:**
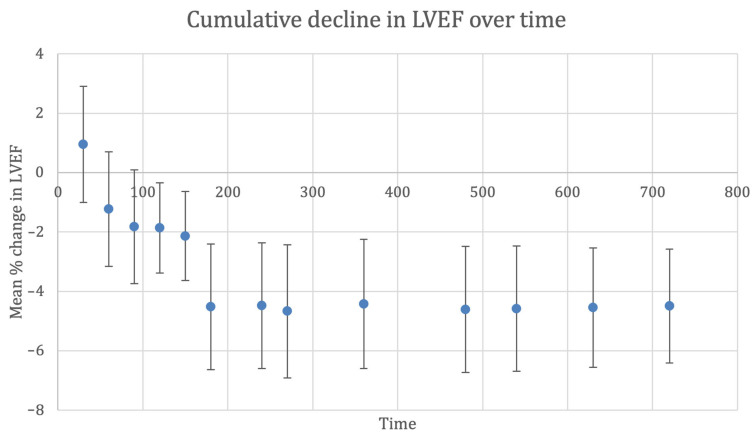
Cumulative decline in left ventricular ejection fraction (%) over time (days). Whiskers represent 95% confidence interval. Abbreviations: CI, confidence interval; LVEF, left ventricular ejection fraction.

We performed a meta-analysis of the pooled mean difference in LVEF following anthracycline-based chemotherapy, as shown in Figure 2 and Table 1. Using a random-effects model, the overall reduction in post-anthracycline pooled mean LVEF in the placebo arms of included randomised controlled trials was 4.5% (95% CI, 2.6 to 6.4). The trend in LVEF showed a progressive decline until approximately 180 days from anthracycline therapy, after which the cumulative decline in LVEF reported across studies was 4.4% to 4.6%. Thereafter, there was no further significant change in mean LVEF beyond 180 days. Only one study used cardiac magnetic resonance imaging to assess LVEF, while all other studies used echocardiography [17,39]. Bosch et al. found that after six months, LVEF significantly decreased in controls, resulting in a −3.1% absolute difference using echocardiography (*p* = 0.035), and −3.4% (*p* = 0.09) in the 59 patients that underwent cardiac magnetic resonance imaging. The PRADA trial by Heck et al. reported a similar overall decline in LVEF of 2.6% (95% CI, 1.5 to 3.8) percentage points in the placebo group.

### 3.3. Sensitivity and Subgroup Analysis 

We undertook several pre-planned subgroup analyses according to key trial, patient and treatment characteristics to identify risk factors and associations for a poorer prognosis. This involved cancer patients receiving over a mean cumulative anthracycline dose (CAD) of 300 mg/mm^2^ for subgroup analysis, as anthracyclines are established to cause cardiotoxicity in a dose-dependent manner [18,24,28,29,31,32,33,34,35,36].

Subgroup analysis was performed according to the mean cumulative anthracycline dose (CAD) which cancer patients received to account for the dose-dependent cardiotoxicity, stratifying studies which reported a mean CAD above 300 mg/m^2^ and studies between 200 to 300 mg/m^2^ in Table S4. A significant difference was observed between studies. Between 180 and up to 540 days of post-anthracycline chemotherapy, a cumulative LVEF decline of 6.2% (95% CI: 2.6 to 9.9) to 6.7% (95% CI: 2.9 to 10.5) was observed in cohorts receiving above 300 mg/m^2^. This was a significantly more profound reduction than in cohorts receiving CAD 200 to 300 mg/m^2^, which had a cumulative LVEF decline of 3.7% (95% CI: 1.9 to 5.5) at 180 days and 3.4% (95% CI: 2.4 to 4.3) at 630 days. Similar to the overall analysis, a plateauing effect was observed in the LVEF decline after at least 180 days.

Further subgroup analyses according to the baseline LVEF prior to anthracycline administration are presented in Table S5, with overall decline remaining similar. We also excluded studies in which more than 10% of the cancer patients received trastuzumab [16] (Table S6), but this did not yield significant differences in the trend.

### 3.4. Risk of Developing Significant Systolic Dysfunction

**Figure 3:**
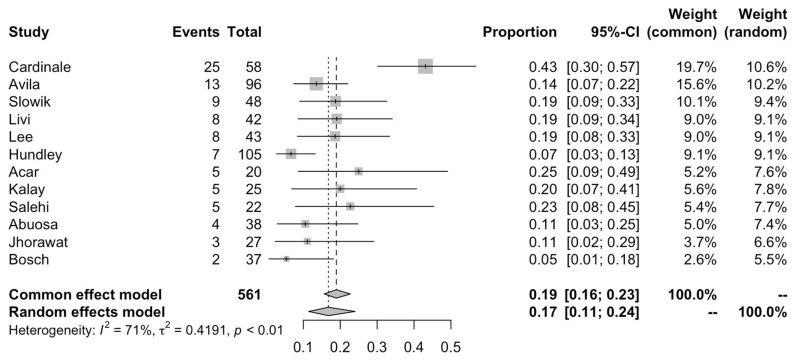
Pooled risk of developing clinically relevant LVEF decline (at least 10% in LVEF from baseline or post-anthracycline LVEF value below 50%).

We extracted the incidence of significant reductions in systolic function as well as their definitions from each cohort. Amongst studies which measured and reported the risk of a 10% decline in LVEF from baseline to a value below 50%, the risk ranged from 7% to as high as 43 (Figure 3). The overall pooled risk was 17% (95% CI: 11 to 24; I2 = 71%) with the random effects model in Figure 3. Outlier analysis identified this heterogeneity to come primarily from the cohort by Cardinale et al. that featured a risk of 43% and 8% (Remaining I2 = 25%). Boekhout et al. used a different LVEF cut-off of 15% below the absolute value of 45%, occurring in 16 of 103 (15.5%) patients receiving anthracyclines without cardioprotection [16]. Georgakopoulos et al. reported the incidence of clinical heart failure in patients that were followed up for a mean of 31 months after receiving anthracyclines, finding it developed in 3 of 40 (7.5%) patients without cardioprotection [22].

### 3.5. Natriuretic Peptides

We performed a meta-analysis of the pooled change in serum levels of natriuretic peptides following anthracycline-based chemotherapy, as shown in Figure S2 and Table S7. Using a random-effects model, the overall pooled change in mean natriuretic peptides in the placebo arms of included randomised-controlled trials was relatively negligible up to 90 days following chemotherapy. From 360 days, a large, but statistically insignificant rise of 8.1 (95% CI: −14.5 to 30.7) was observed. The cumulative rise in natriuretic peptides from baseline up to 630 days (22.1; 95% CI: 2.4 to 41.9) was significant. Overall, the trend in natriuretic peptides observed in cohorts largely mirrored that of the decline in LVEF.

## 4. Discussion

To the best of our knowledge, we present the first systematic review and meta-analysis to evaluate the course and prognosis of cardiac function in patients receiving anthracyclines not receiving cardioprotection and describe the natural effect of therapy. Several large cohort studies have studied the onset of cardiac dysfunction following chemotherapy to months after anthracycline administration [5]. A recent meta-analysis also found similar findings of a lower than previously reported decline in LVEF post anthracycline therapy, but was only limited to a period of six months after therapy without analysing time as a factor, which is crucial given cardiac remodelling [41]. Our review included cohorts which followed patients over two years from the administration of anthracyclines and demonstrated that there was no significant decline after six months. This suggests that cardiotoxicity and associated remodelling is likely to be maximal up to six months following anthracycline administration, and this period may be the most crucial for concurrent cardioprotection to prevent adverse remodelling. The potential confounding effect of receiving concomitant trastuzumab also appeared negligible in comparison to the observed LVEF decline with anthracyclines alone.

It is established in the literature that the cardiotoxic effect of anthracyclines is dose-dependent [42,43]. Our review supports this by providing further evidence that in patients receiving a CAD over 300 mg/m^2^, the mean absolute decline in LVEF after six months may be as high as 10%. Across cohorts, the risk of developing a decline in LVEF over 10% to a value less than 50% in our pooled analysis is approximately one in six.

Various pharmacotherapeutic agents conventionally used in the treatment of heart failure have been explored in randomised-controlled trials for the prevention of cardiac dysfunction, including inhibitors of the renin–angiotensin–aldosterone system and beta-blockers [44]. At present, strategies recommended for the prevention of chemotherapy-induced cardiac dysfunction include optimisation of anthracycline doses, tailoring of antineoplastic regimens depending on patient risk factors and dexrazoxane in those receiving high doses of anthracyclines [44,45]. Our findings also support recommendations on monitoring the frequency of left ventricular function more intensely up to 180 days, after which the intensity of screening may be lowered. Importantly, although there have been recommendations for the initiation of therapies for primary prophylaxis, there is a paucity of evidence on when and how to stop such therapies. Our findings of no significant LVEF decline beyond 180 days may suggest that it is safe to consider weaning patients off such therapies after 180 days from the initiation of anthracycline therapy. 

This would also have implications for the design and defined outcomes of future trials evaluating the efficacy of cardioprotection for anthracyclines. Many trials which utilise the mean decline in LVEF as the primary outcome for trials of primary prophylaxis may find results largely equivocal because the absolute reduction may not be clinically apparent. Additionally, using the definition of clinically relevant cardiac dysfunction of a decline in LVEF of 10% or mean LVEF < 50%, this was observed in approximately one in six patients. Hence, empirical treatment of all patients receiving anthracycline therapy may not be truly effective. Further research should focus on identifying the truly high-risk subset of patients who will benefit most from primary prophylaxis. Overall, cancer patients represent an especially vulnerable population due to both disease and treatment factors [46,47,48]. The prevention of cardiotoxicity is of critical concern given the long-term risks it can have on morbidity, mortality and quality of life [49,50,51]. This highlights the importance of studying the efficacy of drugs with a cardioprotective effect [52,53] and optimising cardioprotective regimens. Aside from pharmacological cardioprotection, lifestyle interventions such as aerobic exercise have been explored in clinical studies. Due to oxidative damage and the induction of pro-inflammatory states having a theorised role in cardiotoxicity, optimising comorbidities such as metabolic diseases that may contribute to systemic inflammation would also be beneficial [54,55,56].

This study faced several limitations. Firstly, while studies evaluated echocardiographic parameters and cardiac biomarkers, few studies have investigated clinical outcomes such as the incidence of heart failure or cardiac mortality. As such, we were unable to correlate our findings with clinical outcomes. Secondly, the lack of baseline characteristics and individual patient available for meta-analysis may have resulted in heterogeneity across studies not being adequately accounted for in our analysis. Details of outcome measurement, such as instrument, calculations and assumptions made in determining the parameters of cardiovascular function were also not explicitly reported in numerous studies. Thirdly, we were unable to account for other existing concurrent therapies such as radiotherapy and immunotherapy which may have been administered due to a lack of reporting. Lastly, a majority of patients did not have a concomitant cardiovascular disease, such as ischemic heart disease. As such, our findings may not be extrapolated to subgroups of cancer patients with significant cardiovascular disease.

## 5. Conclusions

In this systematic review and network meta-analysis, we observed that the mean decline in systolic function amongst patients receiving anthracycline therapy in contemporary clinical practice was 4.5%. Maximal decline in systolic function occurred at 180 days following anthracycline chemotherapy, after which a marked plateauing was observed. Clinically relevant declines in LVEF using a guideline definition occurred in one in six patients. Further research to best identify suitable patients who will benefit most from cardioprotective therapies when receiving anthracyclines is required.

## Figures and Tables

**Table 1:** Cumulative decline in left ventricular ejection fraction (%) over time (days).

Time (Days)	Number of Studies	Mean LVEF Change	Lower CI	Upper CI	*n*
30	2	0.949	−1.008	2.906	45
60	4	−1.229	−3.160	0.702	101
90	8	−1.826	−3.744	0.092	325
120	11	−1.866	−3.384	−0.348	417
150	12	−2.140	−3.639	−0.640	442
180	20	−4.522	−6.638	−2.406	695
240	20	−4.477	−6.594	−2.360	689
270	20	−4.670	−6.907	−2.433	687
360	21	−4.424	−6.600	−2.248	726
480	22	−4.609	−6.728	−2.489	744
540	22	−4.579	−6.691	−2.466	737
630	23	−4.550	−6.558	−2.543	770
720	24	−4.494	−6.415	−2.573	910

Abbreviations: CI, confidence interval; LVEF, left ventricular ejection fraction; *n*, number of patients.

## Data Availability

All analysis was developed using published data. All supplementary material related to this submission is available together with this manuscript.

## Supplementary Materials

The following are available online at https://www.mdpi.com/article/10.3390/cancers15020512/s1, Table S1: Search strategy; Table S2: Characteristics of included studies; Table S3: Details of outcome assessment; Table S4: Subgroup analyses of cumulative decline in left ventricular ejection fraction (%) over time in cohorts of cancer patients receiving a cumulative anthracycline dose above 300 mg/m2 versus a cumulative anthracycline dose between 200 to 300 mg/m^2^; Table S5: Subgroup analyses of cumulative decline in left ventricular ejection fraction (%) over time in cohorts of cancer patients (A) with a baseline LVEF of 65 to 70% (B) with a baseline LVEF of 60 to 65%; Table S6: Subgroup analyses of cumulative decline in left ventricular ejection fraction (%) over time in cohorts of cancer patients, excluding studies with at least 10% of cancer patients receiving trastuzumab; Table S7: Cumulative rise in natriuretic peptides (standardised mean) over time; Figure S1: Risk of bias assessment; Figure S2: Cumulative rise in natriuretic peptides (standardised mean) over time. Whiskers represent 95% confidence intervals.
