# Supplementary material for: Natural Progression of Left Ventricular Function following Anthracyclines without Cardioprotective Therapy: A Systematic Review and Meta-Analysis
Source: Cancers (Basel). 2023 Jan 13;15(2):512. doi: 10.3390/cancers15020512 (PMC9856944; doi:10.3390/cancers15020512)
Supplement: Supplementary file 1 [file cancers-15-00512-s001.zip › cancers-2128650-supplementary.pdf]

Contents:

Table S1: Search strategy
Table S2: Characteristics of included studies
Table S3: Details of outcome assessment
Table S4: Subgroup analyses of cumulative decline in left ventricular ejection fraction (%) over time in cohorts of cancer patients receiving a cumulative anthracycline dose above 300 mg/m2 versus a cumulative anthracycline dose between 200 to 300 mg/m2.
Table S5: Subgroup analyses of cumulative decline in left ventricular ejection fraction (%) over time in cohorts of cancer patients (A) with a baseline LVEF of 65 to 70% (B) with a baseline LVEF of 60 to 65%.
Table S6: Subgroup analyses of cumulative decline in left ventricular ejection fraction (%) over time in cohorts of cancer patients excluding studies with at least 10% of cancer patients receiving trastuzumab.
Table S7: Cumulative rise in natriuretic peptides (standardised mean) over time.
Figure S1: Risk of bias assessment.
Figure S2: Cumulative rise in natriuretic peptides (standardised mean) over time. Whiskers represent 95% confidence intervals.

Table S1. Search strategy.

Database	Search terms	Results
PubMed	(anthracycline or anthra* or epirubicin or doxorubicin or daunorubicin or trastuzumab or herceptin) and (cardiac or cardiotox* or cardiac dysfunction or lvef or ejection fraction or troponin or creatine kinase or natriuretic peptide or bnp or nt-bnp or stress or strain or systolic or diastolic) and (rct OR (random* AND (control*)))	1,440
EMBASE	('anthracycline'/exp OR anthracycline OR anthra* OR 'epirubicin'/exp OR epirubicin OR 'doxorubicin'/exp OR doxorubicin OR 'daunorubicin'/exp OR daunorubicin OR 'trastuzumab'/exp OR trastuzumab OR 'herceptin'/exp OR herceptin) AND ('cardiac'/exp OR cardiac OR cardiotox* OR 'cardiac dysfunction'/exp OR 'cardiac dysfunction' OR (('cardiac'/exp OR cardiac) AND dysfunction) OR lvef OR 'ejection fraction'/exp OR 'ejection fraction' OR (ejection AND fraction) OR 'troponin'/exp OR troponin OR 'creatine kinase'/exp OR 'creatine kinase' OR (('creatine'/exp OR creatine) AND ('kinase'/exp OR kinase)) OR 'natriuretic peptide'/exp OR 'natriuretic peptide' OR (natriuretic AND ('peptide'/exp OR peptide)) OR bnp OR 'nt bnp' OR 'stress'/exp OR stress OR 'strain'/exp OR strain OR systolic OR diastolic) AND (rct OR (random* AND control*))	3,514
CENTRAL	In all fields: (anthracycline or anthra* or epirubicin or doxorubicin or daunorubicin or trastuzumab or herceptin) and (cardiac or cardiotox* or cardiac dysfunction or lvef or ejection fraction or troponin or creatine kinase or natriuretic peptide or bnp or nt-bnp or stress or strain or systolic or diastolic) and (rct OR (random* AND (control*)))	1,072

Table S2. Characteristics of included studies.

Study	Year	n, without cardioprotection	Gender (% male)	Mean age	Cancer	Anthracyclines	Baseline mean LVEF (%)	Duration of follow-up (Days)
Lee	2021	43	0	48.5	Breast	Doxorubicin	64	480
Kaya	2013	18	0	50.5	Breast	Epirubicin	66.6	480
Avila	2018	13	0	52.9	Breast	NR	65.2	180
Bosch	2013	37	NR	50	Haematological cancers	NR	62.59	630
Boekhout	2016	206	0	49	Breast	NR	NR	630
Elitok	2014	40	0	52.9	Breast	NR	65	180
Nabati	2017	40	0	47.1	Breast	Doxorubicin	61.13	180
Acar	2011	20	45	52.6	Non-Hodgkin's lymphoma, multiple myeloma, leukemia	Doxorubicin or idarubicin	62.9	180
Kalay	2006	25	16	49	Breast, lymphomas	Doxorubicin or epirubicin	69.7	180
Georgakopoulos	2010	40	53	49.1	Lymphomas	Doxorubicin	67.6	360
Jhorawat	2016	27	66.7	38.74	Haematological cancers	Doxorubicin	67.56	180

Salehi	2011	22	36.4	43.5	Breast, lymphomas	Doxorubicin or epirubicin	58.56	120
Dessi	2013	24	33.3	53	Various	Epirubicin	66	540
Livi	2021	42	0	48	Breast	NR	64.1	360
Janbabai	2016	35	11.4	47.06	Various	Doxorubicin	59.61	180
Cardinale	2006	58	33	44	Breast, haematological cancers	NR	61.8	270
Abuosa	2018	38	24	40.4	Breast, lymphomas	Doxorubicin	62	180
Cochera	2018	30	0	52	Breast	Doxorubicin	61	126
Sun	2015	40	0	55.11	Breast	Epirubicin	65	126
Slowik	2020	48	0	45	Breast	NR	NR	365
Hundley	2022	140	8	49.4	Breast, lymphomas	Doxorubicin	61.7	720
Esfandbod	2021	30	0	46.2	Breast	Doxorubicin	54.9	360
Wihandono	2021	25	0	50.8	Breast	Doxorubicin	65.64	150
Farahani	2019	35	0	57.4	Breast	Doxorubicin or idarubicin	54.93	90
Cadeddu	2010	24	0.33	53	Various	Epirubicin	66	28
Heck	2021	30	0	51	Breast	Epirubicin	NR	690

Abbreviations: Left ventricular ejection fraction, LVEF; NR, not reported; NS, not studied

Table S3. Details of outcome assessment.

Study	Year	Were outcome assessors blinded?	Method of measuring LVEF	Definition of significant cardiotoxicity	Natriuretic peptide(s) assessed	Was lab measurement standardised for all participants?
Lee	2021	Blinded	Echo	Drop in LVEF >10% below baseline	NS	NS
Kaya	2013	Blinded	Echo	NR	NT-pro-BNP, pmol/L	Yes
Avila	2018	Blinded	Echo	Drop in LVEF >10% below baseline	BNP, pg/mL	Yes
Bosch	2013	Blinded	Echo and CMR	Drop in LVEF ≥10% to a value below 50%	BNP, ng/L	Yes
Boekhout	2016	Blinded	Echo	Drop in LVEF ≥15% or to a value below 45%	NT-pro-BNP, pmol/L	Yes
Elitok	2014	NR	Echo	NS	NS	NS
Nabati	2017	NR	Echo	NS	NS	NS
Acar	2011	Blinded	Echo	Drop in LVEF to a value below 50%	NS	NS
Kalay	2006	NR	Echo	Drop in LVEF to a value below 50%	NS	NS
Georgakopoulos	2010	Blinded	Echo	NS (No LVEF definition used, incidence of heart failure reported)	NS	NS

Jhorawat	2016	Blinded	Echo	Drop in LVEF >10% to a value below 55% if asymptomatic, or >5% to a value below 55% if symptomatic	NS	NS
Salehi	2011	NR	Echo	NR (LVEF definition not specified)	NS	NS
Dessi	2013	Unblinded	Echo	NS	NS	NS
Livi	2021	Blinded	Echo	Drop in LVEF >10% below baseline	NS	NS
Janbabai	2016	Blinded	Echo	Drop in LVEF >10% to a value below 55% if asymptomatic, or >5% to a value below 55% if symptomatic	NS	NS
Cardinale	2006	Blinded	Echo	Drop in LVEF ≥10% to a value below 50%	NS	NS
Abuosa	2018	Blinded	Echo	Drop in LVEF to a value below 50%	NS	NS
Cochera	2018	Unblinded	Echo	NS	NS	NS
Sun	2015	Blinded	Echo	NS	NS	NS
Slowik	2020	Blinded	Echo	Drop in LVEF >10% below baseline	NT-pro-BNP, pg/L	NS
Hundley	2022	Blinded	Echo	Drop in LVEF >10% below baseline	NS	NS

Esfandbod	2021	Blinded	Echo	Any decrease in LVEF below baseline	NS	NS
Wihandono	2021	NR	Echo	NS	NS	NS
Farahani	2019	Blinded	Echo	NR	NS	NS
Cadeddu	2010	NR	Echo	NS	NS	NS
Heck	2021	Blinded	Echo and CMR	Drop in LVEF ≥10% to a value below 53% (Excluded from analysis due to number of events in control not reported)	NT-pro-BNP, pg/mL	Yes

Abbreviations: Left ventricular ejection fraction, LVEF; NR, not reported; NS, not studied; Echo, echocardiography; CMR, cardiac magnetic resonance imaging; NT-pro-BNP, N-terminal pro-brain natriuretic peptide.

Table S4. Subgroup analyses of cumulative decline in left ventricular ejection fraction (%) over time in cohorts of cancer patients receiving a cumulative anthracycline dose above 300 mg/m2 versus a cumulative anthracycline dose between 200 to 300 mg/m2. Abbreviations: k, number of studies; CI, confidence interval; n, number of patients.

	Cohorts with CAD >300 mg/m2					Cohorts with CAD 200-300 mg/m2				
Time (days)	k	Mean	Lower CI	Upper CI	n	k	Mean	Lower CI	Upper CI	n
30	1	2	0.457	3.542	21					
60	1	0	-1.352	1.352	21	2	-2.378	-5.578	0.8217	56
90	2	-3.799	-11.247	3.648	79	2	-2.335	-5.633	0.9623	56
120	5	-2.691	-5.628	0.246	171					
150	6	-3.142	-5.715	-0.568	196					
180	10	-6.240	-9.920	-2.559	326	4	-3.711	-5.495	-1.928	116
270	10	-6.603	-10.507	-2.699	324					
360	10	-6.504	-10.487	-2.521	324					
480						4	-3.513	-5.238	-1.789	116

540	10	-6.708	-10.542	-2.874	324					
630						5	-3.358	-4.343	-2.374	153

Table S5. Subgroup analyses of cumulative decline in left ventricular ejection fraction (%) over time in cohorts of cancer patients (A) with a baseline LVEF of 65 to 70% (B) with a baseline LVEF of 60 to 65%. Abbreviations: k, number of studies; CI, confidence interval; n, number of patients.

A: Subgroup analysis of studies with baseline LVEF 65 to 70%					
Time (Days)	k	Mean	Lower CI	Upper CI	n
30	2	0.949404762	-1.008048981	2.906858505	45
60	3	-0.250495486	-0.99088429	0.489893319	58
90	3	-0.196770938	-0.954200274	0.560658397	58
150	4	-1.415413053	-3.883936479	1.053110373	83
180	6	-5.052824098	-10.29468464	0.189036445	126
360	7	-4.310041025	-8.959094917	0.339012866	166

480	8	-4.908847329	-9.102819917	-0.714874741	184
540	8	-5.161042087	-9.180409349	-1.141674825	184
B: Subgroup analysis of studies with baseline LVEF 60 to 65%					
Time (Days)	k	Mean	Lower CI	Upper CI	n
60	1	-3.96666667	-4.601371777	-3.331961563	43
90	2	-5.730447567	-9.289539721	-2.171355412	101
120	4	-3.20508042	-6.504861931	0.09470109	171
180	9	-4.297650721	-6.785509221	-1.809792221	345
270	9	-4.691378247	-7.678009813	-1.70474668	343
360	9	-4.634908361	-7.657462954	-1.612353767	343
480	9	-4.566757609	-7.599097251	-1.534417966	343
630	10	-4.433753982	-7.151800009	-1.715707955	380

720	11	-4.324949777	-6.782451741	-1.867447813	520

Table S6. Subgroup analyses of cumulative decline in left ventricular ejection fraction (%) over time in cohorts of cancer patients excluding studies with at least 10% of cancer patients receiving trastuzumab. Abbreviations: k, number of studies; CI, confidence interval; n, number of patients.

Analysis of LVEF excluding studies with >10% patients receiving trastuzumab					
Time (Days)	k	Mean	Lower CI	Upper CI	n
30	2	0.949404762	-1.008048981	2.906858505	45
60	4	-1.229147886	-3.160625386	0.702329614	101
90	7	-1.687277745	-3.88180335	0.507247859	224
120	10	-1.769694434	-3.437833119	-0.10155575	316
150	11	-2.079387293	-3.720336488	-0.438438099	341
180	18	-4.720534537	-7.054574792	-2.386494282	551
270	18	-4.934523054	-7.398575287	-2.47047082	549

360	19	-4.702005908	-7.080384254	-2.323627563	589
480	20	-4.890950966	-7.19181087	-2.590091062	607
540	20	-4.992631175	-7.248651301	-2.736611049	607
630	21	-4.906991893	-7.055323215	-2.758660571	644
720	22	-4.829087089	-6.877905291	-2.780268887	784

Table S7. Cumulative rise in natriuretic peptides (standardised mean) over time. Abbreviations: k, number of studies; CI, confidence interval; n, number of patients.

Time (days)	k	Mean change	Lower CI	Upper CI	n
30	1	0	-1.636	1.636	96
60	1	2	-0.114	4.114	96
90	1	-1	-2.710	0.710	96
180	1	-2	-3.710	-0.289	96
360	2	8.114	-14.519	30.749	144

480	3	25.299	-10.014	60.613	162
630	5	22.135	2.383	41.887	284

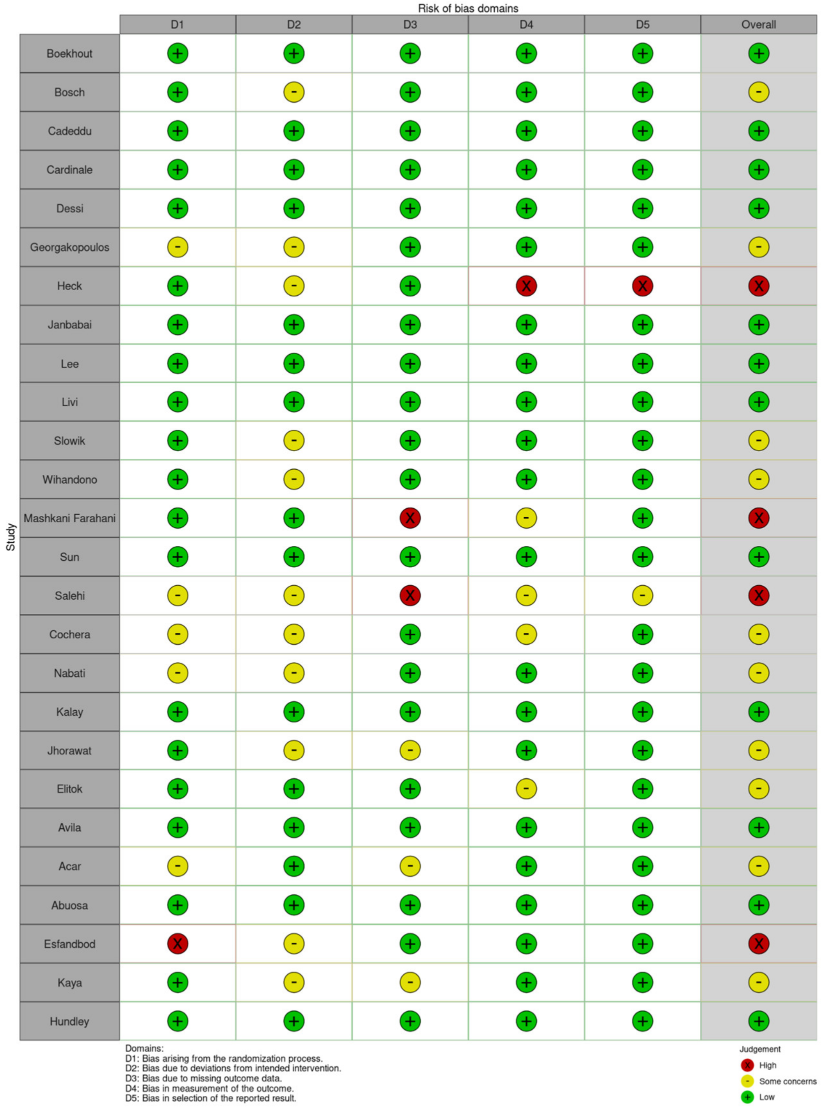

Figure S1. Risk of bias assessment.

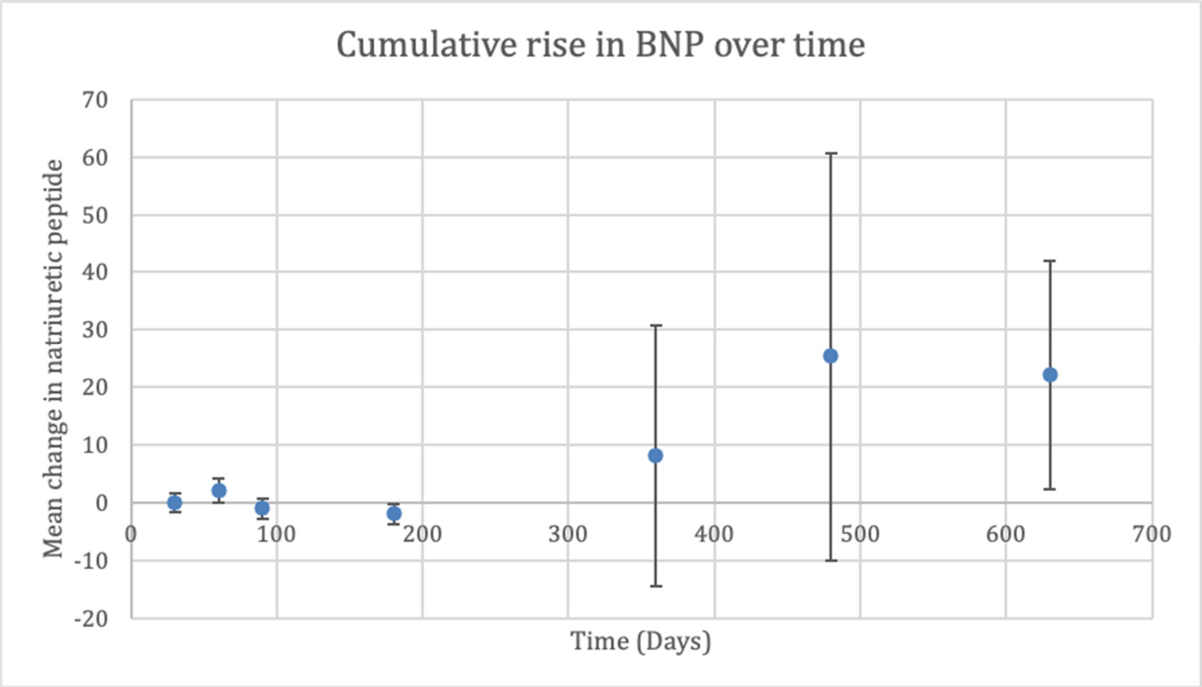

Figure S2. Cumulative rise in natriuretic peptides (standardised mean) over time. Whiskers represent 95% confidence intervals.
